# Cost-Effectiveness of Cetuximab for Advanced Esophageal Squamous Cell Carcinoma

**DOI:** 10.1371/journal.pone.0153943

**Published:** 2016-04-21

**Authors:** Vincent T. Janmaat, Marco J. Bruno, Suzanne Polinder, Sylvie Lorenzen, Florian Lordick, Maikel P. Peppelenbosch, Manon C. W. Spaander

**Affiliations:** 1 Department of Gastroenterology and Hepatology, Erasmus MC-University Medical Center, Rotterdam, The Netherlands; 2 Department of Public Health, Erasmus MC-University Medical Center, Rotterdam, The Netherlands; 3 Third Department of Internal Medicine (Hematology/Medical Oncology), Technical University of Munich, Munich, Germany; 4 University Cancer Center Leipzig (UCCL),Leipzig University,Leipzig, Germany; Brigham and Women's Hospital/Harvard Medical School, UNITED STATES

## Abstract

**Background:**

Costly biologicals in palliative oncology are emerging at a rapid pace. For example, in patients with advanced esophageal squamous cell carcinoma addition of cetuximab to a palliative chemotherapy regimen appears to improve survival. However, it simultaneously results in higher costs. We aimed to determine the incremental cost-effectiveness ratio of adding cetuximab to first-line chemotherapeutic treatment of patients with advanced esophageal squamous cell carcinoma, based on data from a randomized controlled phase II trial.

**Methods:**

A cost effectiveness analysis model was applied based on individual patient data. It included only direct medical costs from the health-care perspective. Quality-adjusted life-years and incremental cost-effectiveness ratios were calculated. Sensitivity analysis was performed by a Monte Carlo analysis.

**Results:**

Adding cetuximab to a cisplatin-5-fluorouracil first-line regimen for advanced esophageal squamous cell carcinoma resulted in an the incremental cost-effectiveness ratio of €252,203 per quality-adjusted life-year. Sensitivity analysis shows that there is a chance of less than 0.001 that the incremental cost-effectiveness ratio will be less than a maximum willingness to pay threshold of €40,000 per quality-adjusted life-year, which is representative for the threshold used in The Netherlands and other developed countries.

**Conclusions:**

Addition of cetuximab to a cisplatin-5-fluorouracil first-line regimen for advanced esophageal squamous cell carcinoma is not cost-effective when appraised according to currently accepted criteria. Cost-effectiveness analyses using outcome data from early clinical trials (*i*.*c*. a phase II trial) enable pharmaceutical companies and policy makers to gain early insight into whether a new drug meets the current eligibility standards for reimbursement and thereby potential admittance for use in regular clinical practice.

## Background

The use of biologicals in palliative oncology is expanding at a rapid pace. These new therapeutic agents may improve patients’ survival and quality of life. However, the money spend on biologicals is expected to increase at a faster rate than the overall spending growth on pharmaceuticals and is projected to represent roughly one fifth of the total costs by 2017 [1]. It would be beneficial for drug companies, policy makers, physicians and patients alike when the cost-effectiveness of these biologicals would become apparent at an early stage of development.

A good example of a carcinoma for which biologicals are being studied in palliative phase II trials is esophageal squamous cell carcinoma (ESCC). ESCC is the predominant form of esophageal carcinoma worldwide, and most patients are diagnosed in advanced stages not amenable to curative treatment [2–4]. Palliative therapy consists of chemotherapy or (chemo-)radiation therapy, management of pain, and achieving optimal nutrition. Currently survival in these patients receiving palliative care remains poor.

Lorenzen et al. conducted a phase II trial in 62 patients with non-resectable epidermal growth factor receptor (EGFR)-expressing ESCC [5]. This trial showed that addition of cetuximab (Erbitux, Merck Serono, Geneva, Switzerland) in palliative treatment of ESCC is likely to prolong survival. The median progression-free survival increased from 3.6 months (95% confidence interval (CI) 1.0–6.2) to 5.9 months (95% CI 3.8–8.0). The median overall survival increased from 5.5 months (95% CI 1.9–9.1) to 9.5 months (95% CI 8.4–10.6). The three adverse events that occurred more often in the cetuximab arm were neutropenia (22% vs 13%), diarrhea (16% vs 0%), and nausea (13% vs 3%), while fatigue decreased (3% vs 10%) [5].

The cost-effectiveness of cetuximab as palliative treatment has not been investigated in ESCC, but has been assessed for other malignancies. Hannouf et al. [6]. estimated that adding cetuximab to platinum-based chemotherapy for squamous cell carcinoma of the head and neck (SCCHN) resulted in a cost-utility ratio of €249,888 per quality adjusted life year (QALY). In addition an incremental cost-effectiveness ratio (ICER) of €242,494 per QALY was reported by the National Institute for Care and health Excellence (NICE) after exploratory analysis using alternative assumptions and parameters in the economic model of the treatment of SCCHN with cetuximab [7]. In metastasized *KRAS* wild type colorectal carcinomas, an ICER of €112,707 per QALY was reported [8]. All these studies with incurable patients receiving cetuximab show ICERs above a maximum willingness to pay threshold of €40,000. This threshold is representative for The Netherlands and other developed countries [9–14].

Phase III trials investigating the effectiveness of adding cetuximab to palliative therapy of ESCC were started after the publication of Lorenzen et al. [15]. Although the addition of cetuximab seems to offer a health benefit, it might have an ICER above the current maximum willingness to pay threshold. Early estimation of the ICER would give early insight into the costs of the therapy regimen and hence the probability that the drug will be reimbursed. If there is too much of a gap between effectiveness and costs, informed decision-making could question the costly development of a drug that will have a hard time to be commercially viable and, importantly, early information sharing would spare the public from developing false expectations.

This paper aims to calculate the expected mean ICER of adding cetuximab to the standard palliative treatment of ESCC in a Dutch health-care setting, based on published data from a phase II trial [5]. It shows that the addition of cetuximab is not cost-effective.

## Methods

### Framework of cost-effectiveness analysis

The cost-effectiveness analysis was based on data from the study performed by Lorenzen et al. [5]. A linear model was used. Two clinical outcome measures, namely mean progression free survival (PFS) and mean overall survival (OS), were used in addition to an approximation of overall costs, in order to calculate ICERs. The cost-effectiveness analysis assumed the health-care perspective, including only direct medical costs. Modelling was done using Microsoft Excel (Microsoft Corporation, Redmond, WA).

### One-way sensitivity analysis

A one-way sensitivity analysis was conducted by taking all input variables of the model and varying them by 10% in both directions. When varying one input variable, other input variables were kept constant.

### Probabilistic sensitivity analysis

The probabilistic sensitivity analysis was performed using a Monte Carlo simulation using Microsoft Excel (Microsoft Corporation, Redmond, WA). For this analysis, 1,000 simulated trials were run, where the values for the OS and PFS of both arms were sampled at random from normalized probability distributions based on the trial sample means and their standard deviation. By using the standard deviation of the mean values the variation of the sample means estimate of the population mean was taken into account. Other parameters of the model were kept constant. This generated the mean costs and mean survival benefit for 1,000 simulated trials.

### Patients

In the study of Lorenzen et al. [5], 62 patients were randomly assigned to the cetuximab, cisplatin, and fluorouracil arm (n = 32) or to the cisplatin and fluorouracil arm (n = 30). The protocol of the study of Lorenzen et al. was approved by the ethics committee for human research at the Technische Universität München, Munich, Germany, and conformed to the principles of the Declaration of Helsinki and its subsequent amendments. The analyses presented in this study fall under the same protocol. All patients gave written informed consent. Patient records/information was anonymized and de-identified prior to analysis. The patients were at least 18 years of age, had histologically confirmed and EGFR-expressing advanced non-resectable ESCC. They had not received (neo)adjuvant chemotherapy within six months of enrollment into the study and had not had prior chemotherapy for recurrent or metastatic disease. Their Eastern Cooperative Oncology Group performance status (ECOG PS) had to be one or less, creatinine clearance had to be above 70 ml/min. They had to have adequate hepatic function, bone marrow function, and a lesion measurable in one dimension of at least one cm in diameter detected by computed tomography (CT) scan was required. Exclusion criteria were a second malignancy, uncontrolled infection, a neuropathy grade more than one, and pregnancy or lactation. Median follow-up time was 21.5 months. Both groups were well balanced with respect to age, tumor differentiation, and intensity of EGFR staining. However, there was a marked difference in the gender balance between the arms with the cetuximab arm containing more female patients. More than 30% of patients in each arm had either prior surgery, radiotherapy, or both. In contrast, only 13% of patients in each arm had received prior systemic chemotherapy [5].

### Therapy regimen

For a maximum of six 29-day cycles, patients received cisplatin 100 mg/m2, day 1, plus fluorouracil 1,000 mg/m2, days 1–5, either alone or in combination with cetuximab. Cetuximab was initially dosed at 400 mg/m2, followed by 250 mg/m2 weekly thereafter [5]. The modelled therapy regimen was constructed to take treatment discontinuation into account, the level of treatment discontinuation was chosen so as to assume a mean cetuximab use equal to that calculated for the phase II trial (see results section).

### Determination of QALYs

**Fig 1:**
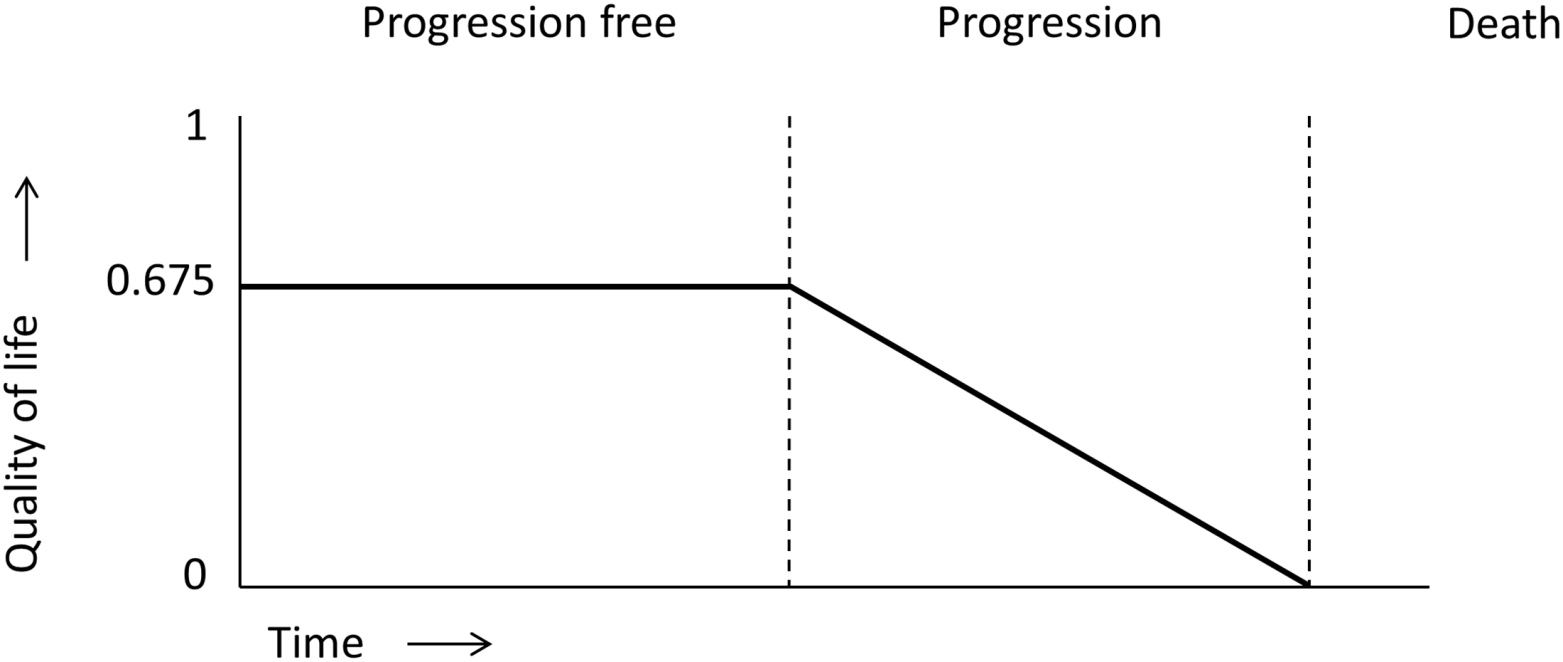
Schematic representation of the health states and utility scores used in the linear model. At diagnosis a utility score of 0.675 was assumed. This utility score remains stable during progression free survival. During progressive disease, it was assumed that the utility score declines linearly from 0.675 to 0 at the time of death.

PFS and OS figures were used from the study of Lorenzen et al. The authors reported median OS and PFS times. Using individual patient data, mean OS and PFS times were calculated, as these data were necessary to calculate the ICER of the proposed therapy regimen. The utility score during PFS was calculated. Asymptomatic adenocarcinoma of the esophagus was associated with a utility score of 0.77, symptomatic adenocarcinoma of the esophagus was associated with a utility score of 0.675 [16, 17]. Since most patients were diagnosed because they presented themselves with symptoms, the utility score of symptomatic disease was used, i.e. 0.675. The utility score during disease progression was defined as the average of the utility score at death, which is zero, and the utility score during PFS. This approach reflects the assumption that the utility score linearly declines from the point of progression to the point of passing away, see Fig 1 for a schematic representation of the assumptions used. QALYs were calculated by: PFS * utility score during PFS + (OS − PFS) * utility score during disease progression: *PFS* × 0.675 + (*OS* − *PFS*) × 0.675. The amount of QALYs gained was calculated by taking the amount of QALYs calculated for the intervention group minus the amount of QALYs calculated for the control group.

### Determination of costs

An estimate was made of the medical resource usage that resulted from the incremental cost of adding cetuximab to the standard treatment. Unless otherwise specified, unit costs were obtained from the Dutch manual for cost-effectiveness research 2010 [18]. The cost of cetuximab for an average patient was calculated. The cost of cetuximab in 2007 was €207 per 100 mg in the Netherlands, and €237,20 in 2009 [19]. The model accounted for: value attributed tax, duration of treatment, the increased dose for the first treatment (400 versus 250 mg), the male female difference in dosage, the relative proportion of both sexes affected by ESCC, and the amount of treatment discontinuation. The cost of an outpatient visit was estimated to be €251 [18]. The number of outpatient clinic visits was calculated. Duration of therapy and the number of outpatient visits that can be combined with visits for chemotherapy were accounted for. The incremental cost of these combined visits was estimated. Lorenzen et al. screened for EGFR expressing tumor patients [5]. Screening for a subset of eligible patients significantly contributes to therapy cost, increasing with decreasing biomarker frequencies [16]. The cost of evaluation of EGFR expression (€750) was based on the reimbursement for such a test in the Dutch healthcare system. It was assumed that 60% of patients have EGFR expressing tumors [20–22]. Although all patients have to be screened. Incremental cost for housing and depreciation of goods (6.5%) and overhead (35.5%) were accounted for. Possible costs associated with an increase in adverse events were not accounted for. All costs and effects were converted to the price level of 2009 according to the general Dutch consumer price index [23]. Results of other studies, reported in CAD and Pound Sterling, were converted to euros using the purchasing power parity index of the year closest to the year in which the study outcome was reported [24]. A cost-effectiveness ratio below €40,000 per gained QALY was assumed to be acceptable. This threshold is representative for the willingness to pay threshold in The Netherlands and other developed countries [9–14]. The time horizon of the base case model was 0.9 years. Because of this short time horizon, neither costs nor clinical outcomes were discounted.

## Results

Lorenzen et al. reported a median OS of 5.5 and a median PFS of 3.6 months for the CF arm as well as a median OS of 9.5 and a median PFS of 5.9 months for the CET CF arm. This translates into a mean OS of 8.6 and a mean PFS of 5.6 months for the CF arm as well as a mean OS of 10.8 and a mean PFS of 7.1 for the CET CF arm. The median cumulative dose of cetuximab per patient was reported to be 8 690mg. The mean cumulative dose of cetuximab per patient was recalculated. This resulted in a mean cumulative dose of 6,443mg cetuximab per patient. Subsequently, the level of treatment discontinuation was chosen so as to assume a mean cetuximab use of 6,444mg.

The mean survival gained by the addition of cetuximab to standard chemotherapy was 0.187 life years and 0.105 QALYs. The mean incremental cost was calculated to be € 26,459 per treated patient. The incremental costs included the costs of cetuximab, additional day treatment facilities, and the cost of evaluation EGFR status, in order of impact. Adding cetuximab to a cisplatin-5-fluorouracil first-line regimen for advanced ESCC resulted in a mean ICER of €252,203 per QALY.

**Fig 2:**
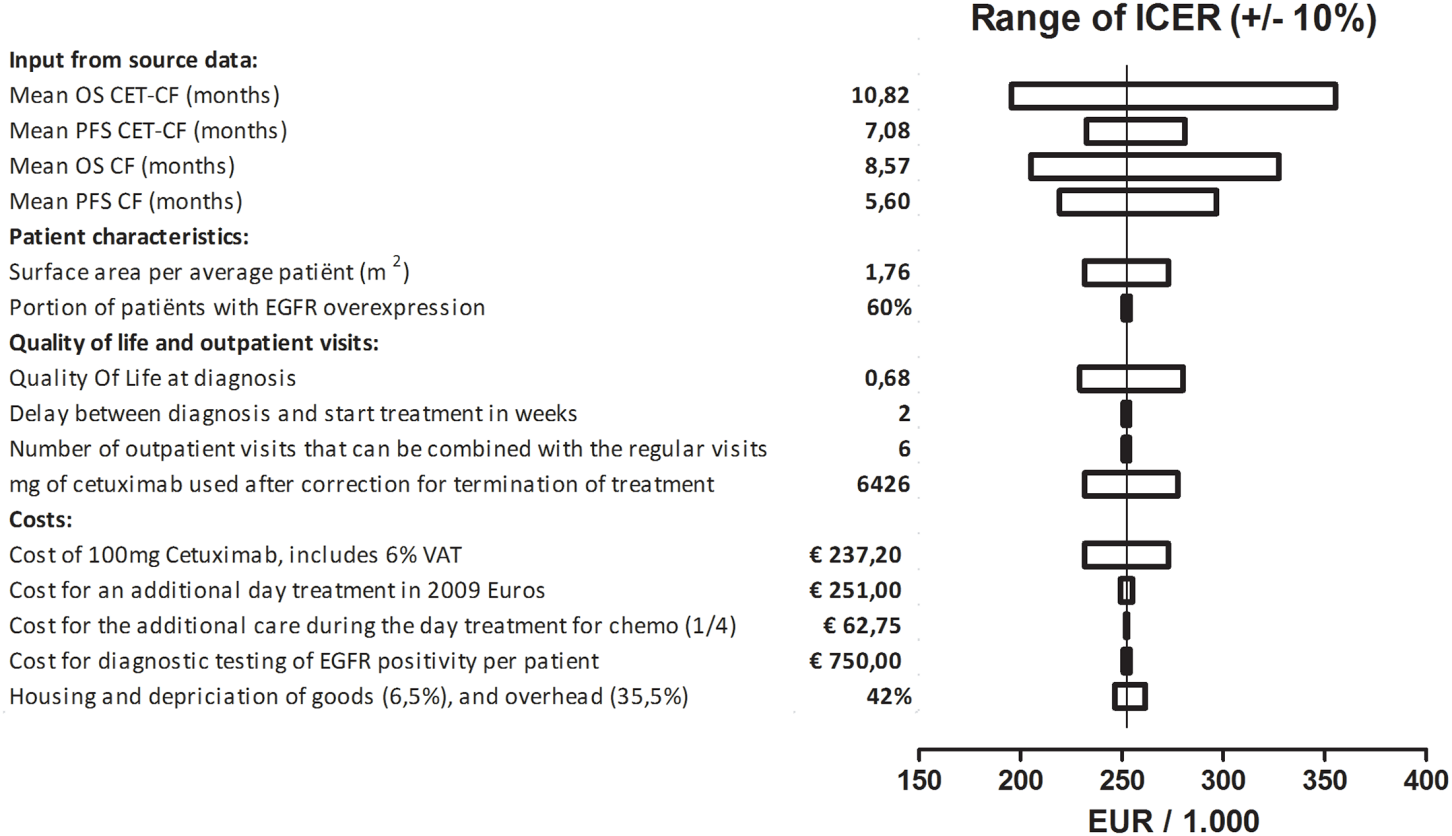
Tornado plot of a one-way sensitivity analysis. Input variables were tested in a one-way sensitivity analysis. These variables were either decreased by 10% of the value used in the base case model, or increased 10% from their original value. The bars represent the range of the ICER if the variable lies between -10% and +10% of its assumed value in the base case model. The ICER form the base case model was indicated by the vertical dotted line.

A one-way sensitivity analysis was conducted by taking all input variables of the model and varying them by 10% in both directions. The resulting ICERs were depicted in a tornado plot, see Fig 2. The input extracted from the phase II trial, had most effect on the determination of the ICER. Other factors of influence were the quality of life assumption used, the amount of cetuximab used, and the price of cetuximab.

**Fig 3:**
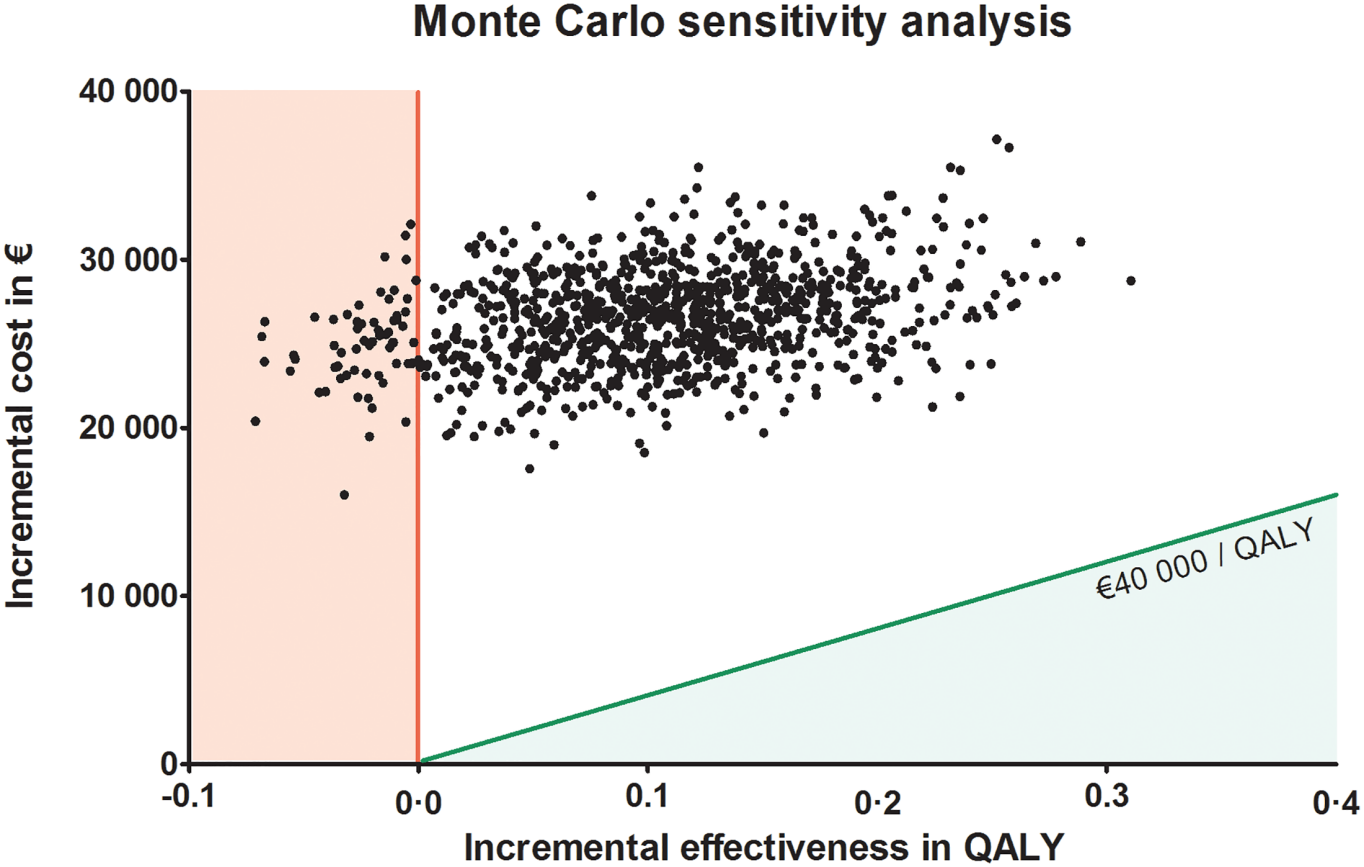
Scatter plot of Monte Carlo sensitivity analysis. One thousand repeated random samplings from the model described are depicted in the figure. Each sampling is depicted by one dot and represents the average QALY gained and the incremental cost of that sampling from the model. The vertical line separates the plane (to the left), for which the intervention is not effective. With the plane to the right for which the intervention is effective. The inclining line depicts the maximum willingness to pay threshold at an ICER of €40,000 per QALY gained. This line separates the plane above it, for which the intervention is effective but not cost effective, with the plane below, for which the intervention is effective and cost effective.

A probabilistic sensitivity analysis was performed by conducting a Monte Carlo analysis, results are shown in Fig 3. Each point represents one of the 1,000 trials runs. The data extracted from the phase II trial had most influence on the determination of the ICER in the one-way sensitivity analysis. Therefore, with respect to the input data for the OS and PFS of both arms, each input was assigned a random value. This was based on the mean and standard deviation extracted from the individual patient data. Other input data was kept constant. The solid diagonal line indicates the €40,000 per QALY gained willingness to pay threshold. Trial points that fall to the left and above this diagonal line indicate a cost-effectiveness of that trial run above the given maximum willingness to pay threshold level. This analysis shows that P < 0.001 of the ICER being below a maximum willingness to pay threshold of €40,000.

## Discussion

The mean ICER of adding cetuximab to a cisplatin-5-fluorouracil first-line palliative regimen for ESCC, is €252,203 per QALY gained. By performing a Monte Carlo analysis we showed that there’s a chance of less than 0.001 that the ICER would be below a threshold of acceptable cost per QALY of €40,000. This threshold is representative for the willingness to pay threshold in the Netherlands. Thus, the therapy regimen will at present not be considered cost-effective by decision makers and, in all probability, will not be reimbursed within the regulated Dutch health insurance system.

In the United Kingdom, the NICE uses an unpublished threshold of £30,000 (€36,997) in 2012 [12, 13]. In Japan the willingness to pay threshold is 5,000,000 JPY (€43,014) in 2010. In the United States of America the threshold is US$ 62,000 (€46,617) in 2010 [14]. This indicates the threshold used, of €40,000, is representative for a willingness to pay threshold used in many developed countries [9, 10]. The price of cetuximab is the dominant driver of the overall costs. Although biosimilars are expected to become available at a 15% to 30% lower prices, substitution of cetuximab with a biosimilar would not make the therapy regimen cost effective [25]. In fact, due to testing of EGFR overexpression and the need for additional day treatments, the ICER was modelled to become €45.907 if cetuximab would be available for free. The ICER that we calculated is in line with previous findings for SCCHN, which has similarities in etiology and pathology, [6, 7, 26] and for *KRAS* wild type colorectal carcinomas [8, 27]. Cost-effectiveness studies after phase II trials have been previously performed [28]. This method has been discussed related to the topic of streamlining the drug development process and early estimation and decision making for reimbursement in 2001 and 2003 [29, 30]. However, no widespread adaptation has taken place.

This study has certain limitations. Quality of life of the patients receiving cetuximab could be affected negatively by side effects. This has not been accounted for in the model. Other limitations of this study are the lack of data on utility scores, both during PFS and after progression of ESCC, therefore data for adenocarcinoma of the esophagus was used. Vial wastage and possible discounts negotiated by hospitals have not been accounted for. Since we did not have access to actual cost data from patient enrolled in this phase II study, another limitation is the use of costs that were taken from a manual for cost-effectiveness.

The results of the phase II trial remain promising despite the considered lack in cost effectiveness. Selecting patients for whom the drug has the most effect will increase treatment effectiveness and remove the burden of side effects from unselected patients. The EGFR staining pattern could be used as a criterion for selection. It has been correlated with poor prognosis in ESCC in a western European population [20]. Tumor specific EGFR downstream signaling mutations in *KRAS*, [8, 31] *BRAF*, [31, 32] *PTEN*, [33] and *PIK3CA* [31, 34] cause reduced benefit of cetuximab therapy in colon carcinoma patients. Germ line polymorphisms could also predict response to treatment, as has been studied in colorectal cancer [35–38]. These options for selection of patients are still speculative and should be investigated further.

## Conclusions

Addition of cetuximab to a cisplatin-5-fluorouracil first-line regimen for advanced ESCC is not cost-effective when appraised according to currently accepted criteria. Cost-effectiveness analyses using outcome data from early clinical trials (*i*.*c*. a phase II trial) enable pharmaceutical companies and policy makers to gain early insights into whether a new drug meets the current eligibility standards for reimbursement and thereby potential allowance for use in clinical practice.

## Supporting Information

S1 Model Cost effectiveness model as described in the manuscript. (XLSX)
